# Predictive Value of the Mayo Adhesive Probability (MAP) Score in Laparoscopic Partial Nephrectomies: A Systematic Review from the EAU Section of Uro-Technology (ESUT)

**DOI:** 10.3390/cancers16081455

**Published:** 2024-04-10

**Authors:** Panagiotis Kallidonis, Theodoros Spinos, Patricia Zondervan, Peter Nyirády, Miguel Ramírez Backhaus, Salvatore Micali, Stephan Hruby, Mario Alvarez-Maestro, Vasileios Tatanis, Evangelos Liatsikos, Ali Serdar Gözen

**Affiliations:** 1 Department of Urology, University of Patras Hospital, 26504 Patras, Greece; thspinos@otenet.gr (T.S.); tatanisbas@gmail.com (V.T.); liatsikos@yahoo.com (E.L.); 2 Laparoscopy Working Group, European Association of Urology (EAU) Section of Uro-Technology; p.j.zondervan@amsterdamumc.nl (P.Z.); nyirady.peter@semmelweis.hu (P.N.); ramirezbackhaus@clinica-urosalud.es (M.R.B.); salvatore.micali@unimore.it (S.M.); stephan.hruby@tauernklinikum.at (S.H.); malvarezmaestro@hotmail.com (M.A.-M.); a.goezen@medius-kliniken.de (A.S.G.); 3 Department of Urology, Amsterdam Medical Centers, 1081 Amsterdam, The Netherlands; 4 Department of Urology, Semmelweis University Budapest, 1083 Budapest, Hungary; 5 Department of Urology, Fundación Instituto Valenciano de Oncología, 46009 Valencia, Spain; 6 Department of Urology, University of Modena and Reggio Emilia, 41121 Modena, Italy; 7 Department of Urology, Tauernklinikum Paracelsusstrasse 8, Zell/See, 5700 Salzburg, Austria; 8 Department of Urology, Hospital Universitario La Paz, 28046 Madrid, Spain; 9 Department of Urology, Medical University of Vienna, 1090 Vienna, Austria; 10 Department of Urology, Medius-Kliniken Ruit, University of Tubingen, 73760 Ostfildern, Germany

**Keywords:** Mayo adhesive probability, MAP, adherent perinephric fat, APF, laparoscopic partial nephrectomy, LPN

## Abstract

**Simple Summary:**

Adherent perinephric fat (APF) represents a challenge for urologists performing partial nephrectomies (PNs). The Mayo Adhesive Probability (MAP) score is a radiographic scoring system which is used for predicting the presence of APF during PNs. The MAP is calculated, taking into consideration two parameters: the posterior perinephric fat thickness and stranding. Although many studies report the ability of the MAP score to predict the presence of APF, there is little evidence regarding the predictive value of the MAP score for various intraoperative and postoperative parameters which are encountered during laparoscopic PNs. This systematic review summarizes all the existing evidence on this topic.

**Abstract:**

The Mayo Adhesive Probability (MAP) score is a radiographic scoring system that predicts the presence of adherent perinephric fat (APF) during partial nephrectomies (PNs). The purpose of this systematic review is to summarize the current literature on the application of the MAP score for predicting intraoperative difficulties related to APF and complications in laparoscopic PNs. Three databases, PubMed, Scopus and Cochrane, were screened, from inception to 29 October 2023, taking into consideration the Preferred Reporting Items for Systematic Reviews and Meta-Analyses (PRISMA) Guidelines. All the inclusion criteria were met by eight studies. The total operative time was around two hours in most studies, while the warm ischemia time was <30 min in all studies and <20 min in four studies. Positive surgical margins, conversion and transfusion rates ranged from 0% to 6.3%, from 0% to 5.0% and from 0.7% to 7.5%, respectively. Finally, the majority of the complications were classified as Grade I-II, according to the Clavien–Dindo Classification System. The MAP score is a useful tool for predicting not only the presence of APF during laparoscopic PNs but also various intraoperative and postoperative characteristics. It was found to be significantly associated with an increased operative time, estimated blood loss and intraoperative and postoperative complication rates.

## 1. Introduction

Partial nephrectomy (PN) represents the gold standard surgical option for localized T1 kidney tumors, irrespective of the surgical approach, according to the European Association of Urology (EAU) Guidelines [1]. Studies comparing open and laparoscopic PN surgical techniques do not report significant differences in surgical, functional and oncological outcomes, while the laparoscopic approach has been shown to present less estimated blood loss (EBL) and a shorter hospital stay [2,3]. The decision to perform a PN is usually made by taking into consideration not only the complexity of the renal mass but also the special anatomy of the surrounding tissues [4]. Renal tumor-specific characteristics evaluated by well-documented tumor morphometric scores, such as the RENAL nephrometry score (RNS) and the PADUA score, have been shown to accurately predict the surgical complexity and the potential complications encountered during PN [5,6]. However, patient-specific characteristics must also be taken into consideration, as significantly affecting dissection gestures and tumor exposure during PNs [7].

**Figure 1:**
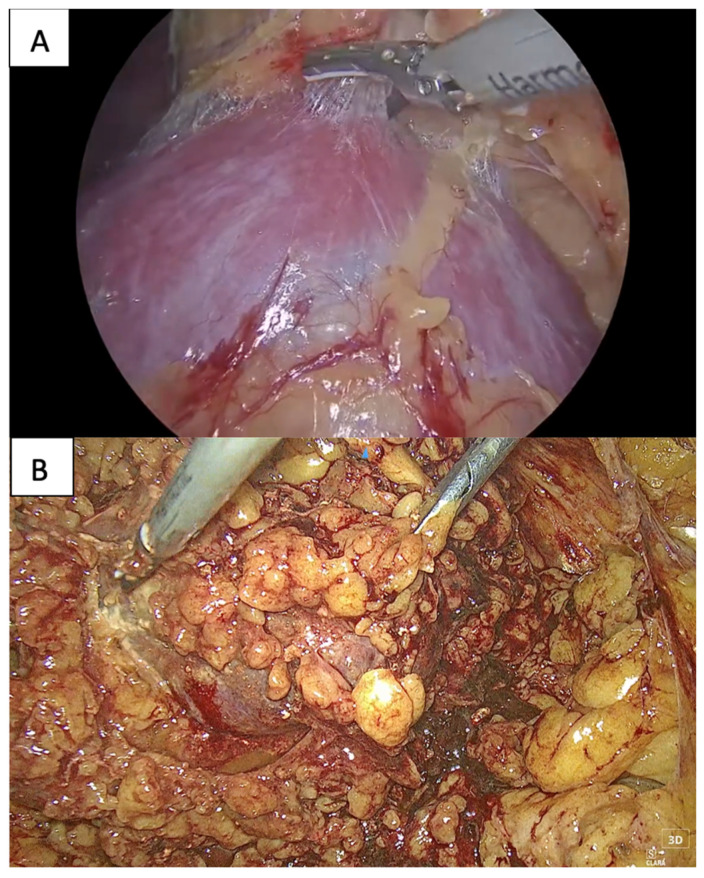
(**A**) During a laparoscopic partial nephrectomy, easy perinephric fat detachment from the kidney is a key to success. (**B**) Otherwise, adherent perinephric fat (APF) is present.

**Figure 2:**
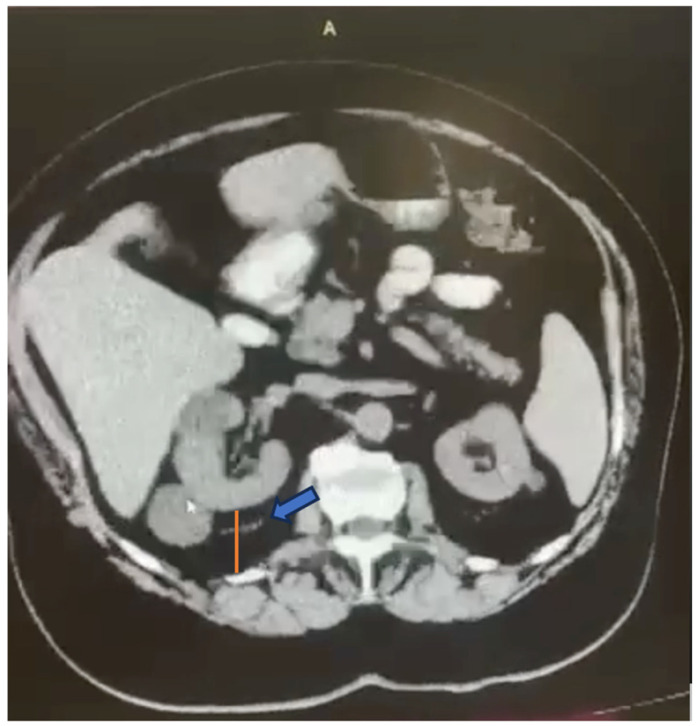
A preoperative scan of a patient with a kidney tumor undergoing laparoscopic partial nephrectomy. The Mayo Adhesive Probability Score takes into consideration the perinephric fat thickness and perinephric stranding. Regarding the perinephric fat thickness, it is calculated with a direct line (red line), at the level of the renal vein, originating from the posterior renal capsule up to the posterior abdominal wall. Perinephric stranding is evidenced as soft tissue attenuation near the kidney, and it is classified according to its severity. In this patient, mild stranding is present (blue arrow). The two scores are then combined into a single MAP score.

Adherent perinephric fat (APF) represents a challenge for urologists performing PNs. APF, also called “sticky fat” or “toxic fat”, is sticky visceral fat, underlying Gerota’s fascia and coming into touch with the kidney parenchyma (Figure 1). APF further complicates kidney mobilization and tumor exposure during PNs, increasing the surgical difficulty of nephron-sparing surgery (NSS) [8,9,10]. The Mayo Adhesive Probability (MAP) score, which was originally described by Davidiuk et al., is a radiographic scoring system which is used for the prediction of the presence of APF during PNs. The MAP is calculated, taking into consideration two parameters: the posterior perinephric fat thickness and stranding [11]. An example of a MAP score calculation is presented in Figure 2. It has been shown to reliably and precisely indicate the presence of sticky fat [8,12,13]. The purpose of this systematic review is to summarize the current literature on the application of the MAP score for predicting intraoperative difficulties related to APF and complications in laparoscopic PNs. Moreover, the MAP score is compared with other nephrometry and radiographic scores, which are used for predicting the intraoperative and postoperative course of patients undergoing PN, such as the RNS and Renal Pelvic Score [14].

## 2. Materials and Methods

### 2.1. Search Strategy

Three databases, PubMed, Scopus and Cochrane, were screened in a systematic manner, from inception to 29 October 2023, taking into consideration the recommendations of the Preferred Reporting Items for Systematic Reviews and Meta-Analyses (PRISMA) Guidelines. This review was performed in accordance with the PRISMA (Preferred Reporting Items for Systematic Reviews and Meta-Analyses) Guidelines. Only human studies and articles in English were accepted. A protocol has been previously registered at https://osf.io on 24 October 2023 (Identifier: DOI 10.17605/OSF.IO/K2V75). Keywords used were as follows: Mayo adhesive probability, MAP, adherent perinephric fat, APF, laparoscopic partial nephrectomy, LPN.

### 2.2. Selection Criteria and Data Extraction

The PICO (patients, intervention, comparison, outcome) criteria were used for defining our search strategy. The patients included were adults (>18 years old) with renal masses undergoing laparoscopic PNs. The intervention should be a laparoscopic PN with a preoperative MAP score calculation. Comparison was made between the MAP score and other nephrometry or radiographic scores, which are used for predicting the feasibility, safety and efficacy of laparoscopic PNs or the presence of adherent perinephric fat. Nevertheless, non-comparative studies were also eligible. The primary outcomes were operative time (OT), estimated blood loss (EBL), conversion to open or radical surgery and positive surgical margins (PSMs). The secondary outcomes were complications according to the Clavien–Dindo Classification System, warm ischemia time (WIT) and hospital stay [15]. Retrospective and prospective studies, both comparative and non-comparative ones, were eligible, while case series were also accepted. Studies assessing the efficacy of the MAP in predicting the presence of APF in general were excluded.

### 2.3. Article Selection

**Figure 3:**
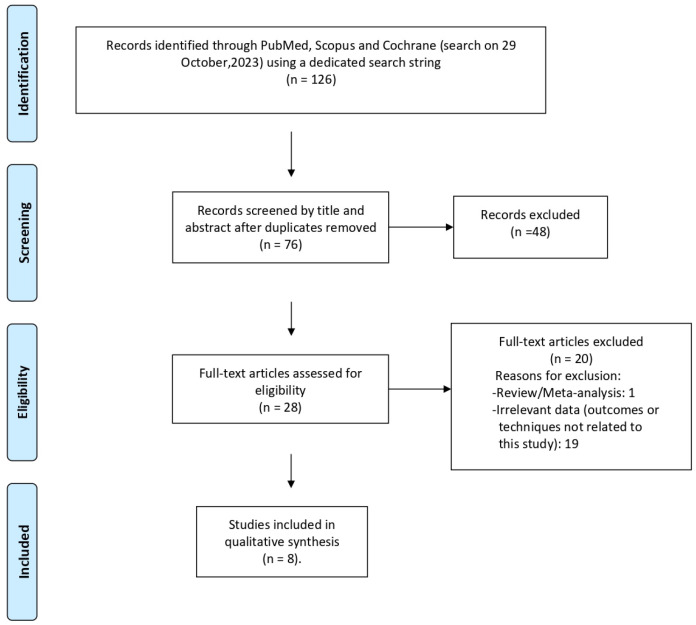
The PRISMA flow chart for the selection of the included studies.

Taking into consideration the PRISMA Guidelines and the predefined inclusion and exclusion criteria, two authors (P.K. and T.S.) reviewed the three databases separately. Any discordance between the two reviewers was resolved by a third independent author (A.S.G.), until a common decision was made. In the beginning, using a dedicated search string, 126 articles were reviewed for eligibility (PubMed: 60, Scopus: 66, Cochrane: 0). When the duplicates were eliminated (*n* = 50), 76 articles were reviewed by title and abstract. After a meticulous review, 48 articles were eliminated, while 28 full-text articles were assessed in a detailed manner by the two authors. Using the SQR3 (survey, question, read, recite and review) technique, 8 relevant studies were deemed eligible and were included in the final qualitative synthesis, while 20 studies were eliminated. The PRISMA flow chart for the selection of the included studies is presented in Figure 3.

## 3. Results

### 3.1. Studies Characteristics

The characteristics of the included studies are presented in Table 1. Eight studies (*n* = 8) were deemed eligible and were included in the final qualitative synthesis [16,17,18,19,20,21,22,23]. The included papers were critically appraised to assess the overall risk of bias by two investigators, using the Quality in Prognosis Studies (QUIPS) tool for non-randomized studies [24]. A study was considered to have a low risk of bias if all six parameters of the QUIPS tool were characterized as at a low risk of bias or if up to one parameter was classified as at a moderate risk of bias. On the contrary, a study was considered to have a high risk of bias if one parameter was classified as at a high risk, of bias or if three parameters or more were classified as at a moderate risk of bias. Finally, a study was considered to have a moderate risk of bias in all other cases [25]. Supplementary Table S1 provides a detailed description of the risk of bias assessment of the included studies. In total, 2205 patients underwent PNs in the selected studies [16,17,18,19,20,21,22,23]. All the studies were retrospective [16,17,18,19,20,21,22,23]. Although none of the studies was a purely comparative one, six out of eight studies included two or more radiographic or nephrometry scores (one of which was the MAP score in all studies) and reported their association with outcomes [17,18,19,20,21,22]. Two studies exclusively reported the association of the MAP with intraoperative and postoperative parameters [16,23]. Most studies reported a retroperitoneal approach for laparoscopic PNs [19,20,21,22], while both transperitoneal and retroperitoneal laparoscopic PNs were reported in three studies [16,18,23]. The most frequently reported score in the comparison group was the RENAL nephrometry score (RNS) [17,18,19,20,21,22].

Yuanxin et al. studied the impact of the MAP score on the surgical complexity of laparoscopic PNs [16], while Hata et al. examined whether the MAP score may predict renal function deterioration after a laparoscopic PN [23]. Bier et al. reported the utility of three different scores (MAP, RNS and Renal Pelvic Score) in predicting postoperative risks after laparoscopic PNs [17]. Yang et al. described a novel nephrometry score, which included the MAP score, and reported its association with perioperative outcomes [19]. Moreover, Jin et al. evaluated the predictive value of the combination of the MAP and RNS scores in predicting intraoperative complications during laparoscopic PNs [20]. With the same rationale, Tan et al. described a novel nomogram, which combines the MAP and RNS scores, and reported its association with intraoperative complications [21]. Qian et al. examined whether the MAP score, along with other factors, could predict the feasibility of segmental artery clamping during a laparoscopic PN [22]. Finally, Fang et al. reported the impact of APF on perioperative outcomes during laparoscopic PNs. This study was included in the final qualitative synthesis, because the authors emphasized the strong association of the MAP score with APF [18].

### 3.2. Patients’ Baseline Characteristics

Table 2 provides a detailed description of the baseline characteristics of the patients who were included in the studies. Most studies reported a slight male predominance, while mean age was uniformly <70 years-old in all included studies [16,17,18,19,20,21,22,23]. Renal masses had a mean size < 4 cm, while most of them were staged as T1a or T1b [16,17,18,19,20,21,22,23]. Four studies reported renal masses which were staged as ≥T2, emphasizing the application of laparoscopic PNs in a wide variety of renal masses, irrespective of size [16,17,18,21]. Most renal masses were Furnham Grade I or II, in the studies which reported relevant data [16,18].

### 3.3. Intraoperative and Postoperative Characteristics

Various intraoperative and postoperative parameters of the included studies are presented in Table 3. The mean total OT was around 2 h in most studies [16,17,18,19,20,21,22,23], while the mean WIT was <30 min in all studies [16,17,18,19,20,21,22] and <20 min in four studies [17,18,20,21]. The PSM, conversion (to open or radical surgery) and transfusion rates ranged from 0% [22] to 6.3% [19], from 0% [22] to 5.0% [21] and from 0.7% (MAP score ≥ 3) [16] to 7.5% [20], respectively. The mean hospital stay ranged from 4 [16] to 8 days [18]. Finally, the majority of the recorded complications were Grade I-II, according to the Clavien–Dindo Classification System [16,17,18,19,20,21,22,23].

**Table 2:** Patients’ baseline characteristics. BMI: body mass index, ASA: American Society of Anesthesiologists Classification System, MAP: Mayo Adhesive Probability Score, RNS: RENAL nephrometry scoring system.

Study Name	Gender	Age	BMI (kg/m^2^)	ASA	Tumor Size (cm)	Side	MAP	RNS	Pathol Ogical Stage	Fuhrman Grade
Yuanxin et al. (2019), [16] (median)	Male 58.7%, Female 41.3% (MAP ≤ 2), Male 88.4%, Female 11.6% (MAP ≥ 3)	51 (43–60) (MAP ≤ 2), 54 (48–62) (MAP ≥ 3)	25.0 (23.0–26.8) (MAP ≤ 2), 26.2 (24.7–29.4) (MAP ≥ 3)	≤2 97.1%, >2 2.9% (MAP ≤ 2), ≤2 93.2%, >2 6.8% (MAP ≥ 3)	2.5 (2–3) (MAP ≤ 2), 2.5 (2–3.2) (MAP ≥ 3)	Left 39.5%, right 60.5% (MAP ≤ 2), left 40.4%, right 59.6% (MAP ≥ 3)	54.1% (MAP ≤ 2), 45.9% (MAP ≥ 3)	6 (5–8) (MAP ≤ 2), 6 (6–8) (MAP ≥ 3)	T1a 93.0%, T1b 5.8%, ≥T2 1.2% (MAP ≤ 2), T1a 95.2%, T1b 2.1%, ≥T2 2.7% (MAP ≥ 3)	1–2 95.6%, 3–4 (4.4%) (MAP ≤ 2), 1–2 90.6%, 3–4 (9.4%) (MAP ≥ 3)
Bier et al. (2017), [17] (mean)	Male 62%, Female 38%	61.7± 12.7	NA	NA	NA	Left 53.9%, right 46.1%	66% (MAP ≤ 2), 34% (MAP ≥ 3)	4–6 69%, 7–9 29%, >9 2%	pT1a 74.8%, pT1b 15.4%, pT2 0.5%, 9.1% pT3a (only RCCs)	NA
Fang et al. (2021), [18] (mean)	Male 64.7%, Female 35.3%	57.1 ± 13.4	24.1 ± 3.7	NA	3.7 ± 1.5	Left 48.8%, right 51.2%	2.0 (0.0, 3.0) median	6.0 (6.0, 8.0) median	pT1a 60.9%, pT1b 30.2%, pT2a 2.8%, ≥pT3 6.1%	I 7.5%, II 75.9%, III 16.6%, IV 0.0%
Yang et al. (2020), [19] (median)	Male 29.6%, Female 70.4%	52 (45–61)	25.1 (22.9–27.2)	Score 1 22.0%, Score 2 73.0%, Score 3 5.0%	29.4 (21.5–36.0) mm	NA	1 (0–3)	8 (6–9)	T1a 83.6%, T1b 16.4%	NA
Jin et al. (2019), [20] (mean)	Male 64.1%, Female 35.9%	54.8 ± 11.9	27.6 ± 5.2	1–2 61.4%, 3–4 48.6%	3.4 ± 0.5	Right 44.6%, left 55.4%	2.08 (IQR 1–3) median	7.06 (IQR 5–9) median	NA	NA
Tan et al. (2021), [21] (mean)	Female 36.3%, Male 63.7%	55.1 ± 11.3	25.2 ± 4.4	1–2 65.9%, 3–4 34.1%	3.4± 1.5	Right 44.7%, left 55.3%	2.04 (IQR, 0–3) median	6.15 (IQR, 5–8)	pT1a 71.3%, pT1b 27.4%, pT2 1.3%	NA
Qian et al. (2019), [22] (mean)	Male 67.6%, Female 32.4%	54.8 ± 12.7	23.0 ± 1.74	NA	3.52 ± 1.48	NA	2 (0–5) median	6 (4–11) median	pT1a 70.7%, pT1B 29.3%	NA
Hata et al. (2021), [23] (mean)	Male 68.3%, Female 31.7% (non-deterioration), Male 73.0%, Female 27.0% (deterioration)	65.5 ± 11.1 (non-deterioration), 66.8 ± 11.5 (deterioration)	24.3 ± 3.81 (non-deterioration), 24.4 ± 2.69 (deterioration)	NA	25.9 ± 11.5 (non-deterioration), 22.3 ± 9.06 (deterioration) mm	Right 58.5%, left 41.5% (non-deterioration), right 54.1%, left 32.4% (deterioration)	2.0 ± 1.7 (non-deterioration), 2.0 ± 1.9 (deterioration) tumor side, 1.4 ± 1.7 (non-deterioration), 2.5 ± 1.8 (non-deterioration) unaffected side.	5.7 ± 1.5 (non-deterioration), 5.6 ± 1.7 (deterioration)	NA	NA

## 4. Discussion

### 4.1. Association of MAP Score with Intraoperative Characteristics

Yuanxin et al. reported that patients with a higher MAP score had a longer total OT and dissection times and increased EBL [16]. Interestingly, the WIT and blood transfusion rates did not differ significantly between the low-MAP and high-MAP groups. According to the authors, the longer OT in the high-MAP group was attributed to a longer dissection time, as long as the APF was more difficult to be peeled off. On the contrary, the WIT recording starts after the clamping of the renal artery, when the APF has already been peeled away [16]. Likewise, Jin et al. showed that the MAP score was correlated with the OT, EBL and intraoperative complications (conversion to radical surgery and injury to adjacent structures) and not with the WIT [20]. The authors reported that the MAP score can reliably predict injury to adjacent organs or major vessels [20]. Although Fang et al. studied the association between the outcomes of laparoscopic PNs and the presence of APF in general, they found that the APF group had a significantly longer OT, greater EBL and higher renal capsule rupture rates. Unexpectedly, the WIT was also found to be longer in the APF group [18]. As already mentioned, this study was included in the final qualitative synthesis, because the association of APF with a high MAP score was emphasized throughout [18]. Similar results have been reported by studies which were not included in this systematic review. Bylund et al. found that patients with ‘sticky fat’ had a significantly longer OT than that of control patients [8], while Kawamura et al. reported that the presence of APF was associated with larger EBL [26]. Finally, Qian et al. reported that the MAP score was an independent predictive factor for the feasibility of segmental artery clamping during laparoscopic PNs, reporting that high MAP scores were associated with the lower viability of segmental renal artery clamping [22]. It is worth mentioning that the MAP score has also been used for predicting the complexity of robotic partial nephrectomies [27]. Ishiyama et al. reported in their single-institute study, involving 311 patients, that a higher MAP score was significantly correlated with a longer dissection time during robotic-assisted partial nephrectomies [27].

### 4.2. Association of MAP Score with Postoperative Characteristics

Bier et al. reported in their study that the MAP score was equivalent with the RENAL score for the prediction of overall complications, according to the Clavien–Dindo Classification (AUC = 0.655) [17]. With a cut-off value of ≥3, the MAP score had a sensitivity of 87.5% for predicting intraoperative and postoperative complications. Nevertheless, the MAP score was not associated with the severity of these complications [17]. Hata et al. investigated the efficacy of the MAP score for the prediction of renal function deterioration after a laparoscopic PN [23]. They reported that the MAP score on the contralateral side, and not on the tumor side, was significantly associated with a loss of renal function postoperatively. In their study, the MAP scores were not always similar between the renal mass side and the contralateral side. However, the authors supported that the MAP score on the contralateral from the tumor side might more precisely describe the PNF environment, because several tumor factors can potentially complicate the MAP score on the affected side [23]. Supplementary Table S2 provides a presentation of the results of the included studies, aggregated with respect to the predefined endpoints. Regarding the intraoperative parameters, the endpoints were OT, dissection time, EBL, intraoperative complications and the feasibility of segmental artery clamping. Regarding the postoperative parameters, the endpoints were postoperative complications according to the Clavien–Dindo Classification and loss of renal function.

### 4.3. Combination of MAP Score with Other Scores

Three studies reported the combination of the MAP score with other radiographic or nephrometric scores, for the prediction of various intraoperative and postoperative parameters. Jin et al. reported that the combination of the MAP score with the RENAL score could more precisely predict the total intraoperative complications which were encountered, when compared with these scores alone [20]. Yang et al. described a novel nephrometry score, the RNP score, which integrates components of both the RENAL and MAP scores, and evaluated its predictive value. In their score, only the R (tumor radius) and the N (nearness to the renal sinus or collecting system) components of the RENAL score, and the posterior perinephric fat thickness component of the MAP score, were taken into consideration [19]. They reported that the RNS score, combining the advantages of both scores, demonstrated a good predictive value, while it showed a better interobserver agreement than the RENAL score [19]. Likewise, Tan et al. developed a novel nomogram for predicting the intraoperative complications which are encountered during laparoscopic PNs [21]. Although their nomogram also used the R and N components of the RENAL score, in contrast with the score described by Yang et al., it included the perirenal fat stranding-type component of the MAP score. According to them, this novel nomogram demonstrated a superior predictive value to the RENAL and MAP scores alone and a comparable predictive value to their combination, though with fewer components [21].

### 4.4. Limitations

The limitation of the current review is the low number of the included studies and their retrospective nature. Moreover, the selected studies presented heterogenous data, and thus, statistical analysis was not possible. Another important limitation is that most studies reported that all procedures were performed by senior surgeons. It is thus possible that surgical experience may be a bias in this survey. However, laparoscopic PN is a challenging laparoscopic operation which is performed by experienced laparoscopic surgeons. Finally, the MAP score, and the other scores compared with it, are subjective in nature, presenting significant interobserver differences.

## 5. Conclusions

The MAP score is a useful tool for predicting not only the presence of APF during laparoscopic PNs but also various intraoperative and postoperative characteristics. In the studies which were included in the final qualitative synthesis, it was significantly associated with a longer total OT, greater EBL and increased intraoperative and postoperative complication rates. Moreover, the limited evidence showed that the MAP score could also predict the feasibility of segmental renal artery clamping and the risk of a postoperative loss of renal function. Further well-designed high-quality studies, such as prospective ones and double-blinded randomized controlled trials (RCTs), are needed so as to draw safer conclusions.

## Figures and Tables

**Table 1:** Studies characteristics. OT: operative time, WIT: warm ischemia time, EBL: estimated blood loss.

Study Name	Journal/Year	Type of Study	Number of Patients	Surgical Technique	Comparison *	Outcomes	Risk of Bias ***
Yuanxin et al. (2019), [16]	Journal of Endourology, 2019	Retrospective	*n* = 318	Transperitoneal/Retroperitoneal	No	OT, dissection time, WIT, EBL.	Low
Bier et al. (2017), [17]	Anticancer Research, 2017	Retrospective	*n* = 280	Not defined	Renal Pelvic Score, RENAL nephrometry score	OT, WIT, complications **	Low
Fang et al. (2021), [18]	World Journal of Surgical Oncology, 2021	Retrospective	*n* = 215	Transperitoneal/Retroperitoneal	RENAL nephrometry score	Surgical approach, OT, WIT, EBL, transfusion, length of postoperative stay, complications **, surgical margin, renal capsule rupture.	Low
Yang et al. (2020), [19]	Chinese Medical Journal, 2020	Retrospective	*n* = 159	Retroperitoneal	RENAL nephrometry score, novel nephrometry score (RNP score)	OT, EBL, WIT, margins and complications **	Low
Jin et al. (2019), [20]	Urologia Internationalis, 2019	Retrospective	*n* = 293	Retroperitoneal	RENAL nephrometry score	OT, WIT, EBL, complications **	Low
Tan et al. (2021), [21]	Investigate and Clinical Urology, 2021	Retrospective	*n* = 637	Retroperitoneal	RENAL nephrometry score	Complications **	Low
Qian et al. (2019), [22]	Laparoscopy and Robotics, 2019	Retrospective	*n* = 225	Retroperitoneal	RENAL nephrometry score	Feasibility of segmental artery clamping	Low
Hata et al. (2021), [23]	International Urology and Nephrology, 2021	Retrospective	*n* = 78	Transperitoneal/Retroperitoneal	No	Postoperative renal function deterioration	Low

* Comparison between different scores. ** Complications according to Clavien–Dindo Classification System. *** According to the Quality in Prognosis Studies (QUIPS) tool for non-randomized studies.

**Table 3:** Intraoperative and postoperative characteristics. OT: operative time, EBL: estimated blood loss, WIT: warm ischemia time, PSM: positive surgical margins.

Study Name	OT (min)	Dissection Time (min)	EBL (mL)	WIT (min)	PSM	Conversion	Hospital Stay (Days)	Complications *	Blood Transfusion
Yuanxin et al. (2019), [16] (median)	110 (90–141) MAP score ≤ 2, 131 (110–158) MAP score ≥ 3	54 (43–74) MAP score ≤ 2, 71 (58–93) MAP score ≥ 3	20 (20–50) MAP score ≤ 2, 50 (20–50) MAP score ≥ 3	20 (15–27) MAP score ≤ 2, 21 (15–26) MAP score ≥ 3	1.2% MAP score ≤ 2, 0% MAP score ≥ 3	0% (radical or open) MAP score ≤ 2, 0.7% MAP score ≥ 3	4 (3–5) MAP score ≤ 2, 4 (3–5) MAP score ≥ 3	≤II 2.3% MAP score ≤ 2, >II 0% MAP score ≥ 3, ≤II 3.4% MAP score ≤ 2, >II 0% MAP score ≥ 3,	1.2% MAP score ≤ 2, 0.7% MAP score ≥ 3
Bier et al. (2017), [17] (mean)	132.9 ± 48	NA	NA	14.5 ± 11	1.79%	NA	7 ± 4	I 0%, II 3.6%, III 9.4%, IV and V 0%	NA
Fang et al. (2021), [18] (mean)	130.7 ± 41.0	NA	50.0 (30.0, 100.0) median	14.3 ± 7.3	2.3%	NA	8.0 (7.0, 9.0) median	I–II 29.8%, III–IV 1.8%	3.3%
Yang et al. (2020), [19] (median)	149 (116–186)	NA	20 (10–50)	25 (18–30)	6.3%	NA	NA	I 34.0%, II 3.9%, III/IV 0%	NA
Jin et al. (2019), [20] (mean)	104.6 ± 43.4	NA	202 ± 156	18.7 ± 5.5	1.4%	4.7% (radical), 1.0% (open)	5.8 ± 2.6	I 0%, II 3.4%, III 6.1%, IV 1.0%	7.5%
Tan et al. (2021), [21] (mean)	111.3 ± 38.7	NA	157.6 ± 63.2	15.3 ± 6.1	NA	5.0% (radical or open)	5.1 ± 2.8	Overall postoperative complications 9.6%	6.6%
Qian et al. (2019), [22] (mean)	87.16 ± 10.59 segmental artery clamping, 92.29 ± 15.92 main artery clamping	NA	214.63 ± 120.19 segmental artery clamping, 170.29 ± 99.93 main artery clamping	27.16 ± 6.01 Segmental artery clamping 29.03 ± 6.28 main artery clamping	0%	0% (open or radical), 15.6% (main renal artery clamping)	NA	I 7.11%, II 6.66%, IIIa 3.1%	6.66%
Hata et al. (2021), [23] (mean)	NA	NA	NA	NA	NA	NA	NA	NA	NA

* According to Clavien–Dindo Classification System.

## Data Availability

The data presented in this study are available on reasonable request from the corresponding author.

## Supplementary Materials

The following supporting information can be downloaded at: https://www.mdpi.com/article/10.3390/cancers16081455/s1, Table S1: Risk of Bias Assessment of included studies according to the Quality in Prognosis Studies (QUIPS) tool for non-randomized studies. Table S2: Predictive value of the MAP score. MAP: Mayo Adhesive Probability, OT: Operative Time, EBL: Estimated Blood Loss, APF: Adherent Perinephric Fat, PN: Partial Nephrectomy.
