# Supplementary material for: Predictive Value of the Mayo Adhesive Probability (MAP) Score in Laparoscopic Partial Nephrectomies: A Systematic Review from the EAU Section of Uro-Technology (ESUT)
Source: Cancers (Basel). 2024 Apr 10;16(8):1455. doi: 10.3390/cancers16081455 (PMC11048046; doi:10.3390/cancers16081455)
Supplement: Supplementary file 1 [file cancers-16-01455-s001.zip › cancers-2935520-supplementary.pdf]

Table S1. Risk of Bias Assessment of included studies according to the Quality in Prognosis Studies (QUIPS) tool for non-randomized studies.

Biases	Yuanxin et al., (2019) [16]	Bier et al. (2017), [17]	Fang et al. (2021), [18]	Yang et al. (2020), [19]	Jin et al. (2019), [20]	Tan et al. (2021), [21]	Qian et al. (2019), [22]	Hata et al. (2021), [23]
**Study Participation**	Low	Low	Low	Low	Low	Low	Low	Low
**Study Attrition**	Low	Low	Low	Low	Low	Low	Low	Low
**Prognostic Factor Measurement**	Low	Low	Low	Low	Low	Low	Low	Low
**Outcome Measurement**	Low	Low	Low	Low	Low	Low	Low	Low
**Study Confounding**	Low	Moderate	Moderate	Moderate	Moderate	Moderate	Moderate	Moderate
**Statistical Analysis and Reporting**	Low	Low	Low	Low	Low	Low	Low	Low
**Overall Risk of Bias**	**Low**	**Low**	**Low**	**Low**	**Low**	**Low**	**Low**	**Low**

Table S2. Predictive value of the MAP score. MAP: Mayo Adhesive Probability, OT: Operative Time, EBL: Estimated Blood Loss, APF: Adherent Perinephric Fat, PN: Partial Nephrectomy.

Study Name	Intraoperative Parameters	Postoperative Parameters
**Yuanxin et al. (2019), [16]**	Patients with a higher MAP score had longer total OT and dissection times and increased EBL.	A statistically significant association was not reported.
**Bier et al. (2017), [17]**	With a cut-off value of ≥3, the MAP score had a sensitivity of 87.5% for predicting intraoperative and postoperative complications. Nevertheless, the MAP score was not associated with the severity of these complications	MAP score was equivalent with the RENAL score for predicting the overall complications, according to Clavien-Dindo Classification (AUC=0.655).
**Fang et al. (2021), [18]**	APF group had significantly longer OT, greater EBL and higher renal capsule rupture rates.	A statistically significant association was not reported.
**Yang et al. (2020), [19]**	A statistically significant association was not reported.	A statistically significant association was not reported.
**Jin et al. (2019), [20]**	MAP score was correlated with OT, EBL and intraoperative complications (conversion to radical surgery and injury to adjacent structures)	A statistically significant association was not reported.
**Tan et al. (2021), [21]**	A statistically significant association was not reported.	A statistically significant association was not reported.
**Qian et al. (2019), [22]**	MAP score was an independent predictive factor for the feasibility of segmental artery clamping during laparoscopic PN.	A statistically significant association was not reported.
**Hata et al. (2021), [23]**	A statistically significant association was not reported.	MAP score on the contralateral side, and not on the tumor side, was significantly associated with loss of renal function postoperatively.
